# Redox Balance, Antioxidant Defense, and Oxidative Damage in the Hypothalamus and Cerebral Cortex of Rats with High Fat Diet-Induced Insulin Resistance

**DOI:** 10.1155/2018/6940515

**Published:** 2018-09-06

**Authors:** Mateusz Maciejczyk, Ewa Żebrowska, Anna Zalewska, Adrian Chabowski

**Affiliations:** ^1^Department of Physiology, Medical University of Bialystok, Mickiewicza 2c Str., 15-222 Bialystok, Poland; ^2^Department of Restorative Dentistry, Medical University of Bialystok, 15-276 Bialystok, Sklodowskiej M.C. 24a Str., 15-274 Bialystok, Poland

## Abstract

Oxidative stress is a key pathogenic factor in both neurogenerative and metabolic diseases. However, its contribution in the brain complications of insulin resistance is still not well understood. Therefore, the aim of this study was the evaluation of redox homeostasis and oxidative damage in the hypothalamus and cerebral cortex of insulin-resistant and control rats. 16 male Wistar rats were divided into two equal groups (*n* = 8): the control and high fat diet group (HFD). Prooxidant enzymes (xanthine oxidase and NADPH oxidase); enzymatic and nonenzymatic antioxidants [glutathione peroxidase (GPx), glutathione reductase (GR), catalase (CAT), superoxide dismutase-1 (SOD-1), and uric acid (UA)]; and oxidative damage products [advanced glycation end products (AGE), 4-hydroxynonenal (4-HNE), malondialdehyde (MDA), and 8-hydroxy-2′-deoxyguanosine (8-OHdG)] as well as the total antioxidant capacity (TAC), total oxidant status (TOS), oxidative stress index (OSI), and total ferric reducing ability of sample (FRAP) were evaluated in the hypothalamus and cerebral cortex as well as serum/plasma of HFD-fed and control rats. The activity of prooxidant enzymes was significantly increased in the cerebral cortex and hypothalamus of HFD-fed rats vs. control rats. Additionally, we have showed enhanced antioxidant efficiency in the hypothalamus (↑CAT, ↑UA, ↑TAC, and ↑FRAP) and cerebral cortex (↑GPx, ↑CAT, ↑SOD-1, ↑UA, ↑TAC, and ↑FRAP) of HFD-fed rats. All of the oxidative damage markers (AGE, 4-HNE, MDA, 8-OHdG, and OSI) were significantly increased in the cerebral cortex of insulin-resistant rats, while only 4-HNE and MDA were markedly higher in the hypothalamus of the HFD group. Summarizing, the results of our study indicate an adaptive brain response to the increased production of free radicals under insulin resistance conditions. Despite the increase in antioxidative defense systems, this mechanism does not protect both brain structures from oxidative damages. However, the cerebral cortex is more susceptible to oxidative stress caused by HFD.

## 1. Introduction

Type 2 diabetes (T2DM, OMIM #125853) is one of the most common metabolic disorders referred to as “the epidemic of the 21st century” [1, 2]. The pathogenesis of T2DM involves two basic pathological defects: impaired insulin secretion and insulin resistance (IR), which may be defined as reduction or lack of insulin sensitivity to the target tissues, such as adipose tissue, muscles, and the liver [3]. An important role in the pathogenesis of IR is attributed to free fatty acids (FFA) derived from a high fat diet (HFD). Excessive accumulation of FFA in cells inhibits glucose uptake, which is responsible for the malfunctioning of signal transduction pathways regulated by diacylglycerol (DAG) [3]. In these conditions, the increased activity of stress-induced serine-threonine kinases (e.g., JNK kinase) leads to phosphorylation of serine residues in the insulin receptor substrate (IRS) which blocks the effect of the insulin signaling pathway [4, 5]. It has been proven that diet-induced IR impairs body functioning and significantly increases a risk of cardiovascular disease, hypertension, cancer, and osteoarthritis [4, 5]. However, more and more attention is paid to the possible complications of IR to the central nervous system (CNS) [6–8]. Although for many years the brain was thought to be a typical non-insulin-dependent organ, recent studies have indicated that insulin can cross the blood-brain barrier (BBB) and it is produced within the brain structures including the hypothalamus, cerebral cortex, cerebellum, amygdala, and hippocampus [8, 9]. In addition, the brain insulin activity is associated with regulation of neurotransmitter/neuromodulator secretion as well as acting a major role in synaptic plasticity, memory, learning processes, and neuronal apoptosis [9, 10]. The latest epidemiological studies have also shown the relationship between systemic IR and dementia, neuroinflammation, depression, or increased incidence of the Alzheimer's disease [6–8]. Although these disorders are characterized by diverse clinical picture, it is suggested that they can be explained by similar mechanisms of cerebral neurodegeneration [7].

A significant role in etiology of IR is also attributed to oxidative stress [11, 12]. Generally, oxidative stress is lack of balance between the formation of reactive oxygen species (ROS) and efficiency of enzymatic and nonenzymatic antioxidative systems [13]. This results in oxidative damage to cell components and thus leads to the impairment of cell structures and biological functions [14]. It is assumed that (in both target organs and the brain) the formation of oxidative stress is related to the increase in oxidation of FFA and glucose, as well as increased generation of oxygen free radicals in the mitochondrial respiratory chain [12, 15]. Additionally, the overproduction of ROS may be caused by the increased glycolysis under hyperglycemic conditions as well as intensification of nonenzymatic glycation (glycosylation) of cellular proteins [16]. The resulted oxidative products, especially advanced glycation end products (AGE), play a key role in the cerebral neurodegeneration [8, 16].

Despite a few reports about the role of oxidative stress in the brain complications of IR, the exact kind of brain oxidative damage is still unknown. There is also a lack of data comparing HFD-related oxidative stress in various brain structures, and thus, the aim of this study was the evaluation of redox homeostasis, enzymatic and nonenzymatic brain antioxidants, and oxidative damage in the different brain structures involved in energy homeostasis (hypothalamus) and cognition processes (cerebral cortex) of HFD-fed rats. This is also the first study that has compared oxidative stress on both systemic (serum/plasma) and local (brain) levels in HFD-induced IR.

## 2. Materials and Methods

### 2.1. Animals

The protocol of the study was approved by the Local Ethical Committee for Animal Experiments of the Medical University of Bialystok, Poland (permission number 89/2015, 2015/109).

The research was conducted on male Wistar rats (cmdb outbred, 5 weeks of initial age, 50–70 g of initial weight). Throughout the whole experiment, a stable temperature (20–21°C ± 2°C), humidity (40–60%), twenty-four-hour rhythm (12 h light/12 h dark cycle), and free access to food and drinking water were preserved. The rats were housed individually in standard cages and remained in constant eye contact with each other. After one week of adaptation, the rats were randomly divided into two equal groups:

1. Control (C) (*n* = 8)
2. High fat diet group (HFD) (*n* = 8)

The animals assigned to the control group received a standard laboratory rat chow containing 13.5 kcal% fat, 24 kcal% protein, and 62.5 kcal% carbohydrates (Agropol, Motycz, Poland) for a period of 8 weeks. The animals from the experimental group were fed HFD containing 59.8 kcal% fat, 20.1 kcal% protein, and 20.1 kcal% carbohydrates (Research Diets Inc., cat no. D12492) also for 8 weeks. Food consumption and body weights were monitored every 3 days. Body mass index (BMI) was also analyzed using the weight and the height (the length from the tip of the nose to the anus). BMI was calculated using the formula: BMI = body weight (g)/length^2^ (cm^2^) [17]. BMI between 0.45 g/cm^2^ and 0.68 g/cm^2^ were assumed to be normal values, whereas obesity was defined as BMI greater than 0.68 g/cm^2^ [17].

After the 8 weeks of experiment and after an overnight fasting, animals were anesthetized by intraperitoneal injection with sodium phenobarbital in a dose of 80 mg/kg BW. Fasting tail-blood glucose analysis was done (Accu-Chek Active Blood Glucose Meter, Roche, Bayer, Germany), and blood samples were collected from the abdominal aorta. The samples were placed into glass tubes (to obtain serum) and glass tubes containing sodium heparin (to obtain plasma). The blood was then centrifuged (3000 × g, 4°C, 10 min), and to protect against sample oxidation and proteolysis, the antioxidant butylated hydroxytoluene (0.5 M solution in acetonitrile, 10 *μ*L/1 mL; Sigma-Aldrich, Germany) and protease inhibitor (Complete Mini, Roche, France) were added [18]. Serum and plasma samples were precooled in liquid nitrogen and stored at −80°C until use.

The hypothalamus and cerebral cortex were collected by one and the same lab technician. The brain structures were freeze-clamped with aluminum tongs, precooled in liquid nitrogen, and stored at −80°C until assays. On the day of biochemical analysis, brain tissues were slowly thawed at 4°C, divided into small pieces, and diluted (1 : 15, *w* : *v*) in ice-cold phosphate buffered saline (PBS; 0.02 M, pH 7.0). To all samples, antioxidant butylated hydroxytoluene and protease inhibitor were added [2]. The brain samples were homogenized on ice (glass homogenizer; Omni TH, Omni International, Kennesaw, GA, USA) and sonicated (1800 J/sample, 20 sec × 3; ultrasonic cell disrupter, UP 400S, Hielscher, Teltow, Germany). Next, the homogenates were centrifuged (5000 × g, 4°C, 20 min), and the resulting supernatants were analyzed on the same day.

Plasma FFA were determined by gas chromatography (GC) [19]. Fasting insulin concentration was assessed by the ELISA method using commercial kit Insulin Rat ELISA BioVendor (Brno, Czech Republic). To confirm IR, the insulin sensitivity was calculated using the homeostasis model assessment of insulin resistance (HOMA-IR) = fasting insulin (U/mL) *×* fasting glucose (mM)/22.5.

### 2.2. Biochemical Assays

The performed analysis included

- determination of prooxidant enzymes—NADPH oxidase (NOX; EC 1.6.3.1) and xanthine oxidase (XO; EC 1.17.3.2)
- determination of enzymatic and nonenzymatic antioxidants—glutathione peroxidase (GPx; EC 1.11.1.9), glutathione reductase (GR; EC 1.8.1.7), catalase (CAT; EC 1.11.1.6), Cu-Zn-superoxide dismutase-1 (SOD-1; EC 1.15.1.1), and uric acid (UA)
- determination of total antioxidant/oxidant status—total antioxidant capacity (TAC), total oxidant status (TOS), oxidative stress index (OSI), and the total ferric reducing ability of sample (FRAP)
- determination of oxidative damage products—advanced glycation end products (AGE), 4-hydroxynonenal (4-HNE) protein adducts, malondialdehyde (MDA), and 8-hydroxy-2′-deoxyguanosine (8-OHdG)

All assays were performed in homogenates of the brain samples (hypothalamus and cerebral cortex). Additionally, enzymatic antioxidants (GPx, GR, CAT, and SOD-1) were evaluated in the serum samples, while another redox/oxidative stress biomarkers in rat plasma samples. Absorbance/fluorescence was measured using microplate reader Infinite M200 PRO Multimode Tecan (Tecan Group Ltd., Männedorf, Switzerland). All determinations were performed in duplicate samples (except for CAT, SOD-1, TAC, and TOS; see below), and all biochemical reagents were obtained from Sigma-Aldrich Germany/Sigma-Aldrich USA. The final results were standardized to one milligram of the total protein. The total protein content was estimated in duplicate samples using the colorimetric bicinchoninic acid (BCA) assay with bovine serum albumin as a standard (Pierce BCA Protein Assay Kit, Rockford, USA) [20].

### 2.3. Prooxidant Enzymes

Activity of prooxidant enzymes (NOX and XO) was assayed immediately after sample collection. NOX activity was analyzed by the luminescence assay using lucigenin as a luminophore [21]. One unit of NOX activity was defined as the amount of enzyme required to release 1 nmol of superoxide anion per one minute. XO activity was evaluated colorimetrically according to Prajda and Weber [22] by measuring the increase in absorbance of uric acid (UA) at 290 nm. One unit of XO activity was defined as the amount of enzyme required to release 1 *μ*mol of UA per one minute.

### 2.4. Enzymatic and Nonenzymatic Antioxidants

GPx activity was assayed spectrophotometrically based on the conversion of NADPH to NADP^+^ [23]. The absorbance was analyzed at 340 nm. It was assumed that one unit of GPx activity catalyzes oxidation of one millimole NADPH for one minute.

CAT activity was estimated spectrophotometrically by measuring the decomposition rate of hydrogen peroxide (H_2_O_2_) at 340 nm [24]. It was assumed that one unit of CAT activity degrades 1 micromole of H_2_O_2_ for one minute. CAT activity was analyzed in triplicate samples.

SOD-1 activity was assayed spectrophotometrically by measuring the cytosolic activity of superoxide dismutase by inhibiting oxidation of epinephrine to adrenochrome [25]. The absorbance was measured at 340 nm. One unit of SOD-1 activity was defined as the amount of enzyme which inhibits oxidation of epinephrine by 50%. SOD-1 activity was estimated in triplicate samples.

UA concentrations were estimated using the commercial colorimetric kit QuantiChrom™Uric Acid Assay Kit DIUA-250 (BioAssay Systems, Hayward, CA, USA). The absorbance was measured at 490 nm.

### 2.5. Total Antioxidant/Oxidant Status

TAC content was determined in triplicate samples using a colorimetric method with 2,2-azinobis-3-ethylbenzothiazoline-6-sulfonic acid radical cation (ABTS^∗+^) [26]. The absorbance was measured at 660 nm, and TAC was calculated from the calibration curve for Trolox (6-hydroxy-2,5,7,8-tetramethylchroman-2-carboxylic acid). TOS content was analyzed bichromatically at 560/800 nm based on oxidation of Fe^2+^ to Fe^3+^ ions in the presence of the oxidants contained in the samples [27]. TOS was estimated in triplicate samples and expressed as *μ*mol H_2_O_2_ equiv./L. Oxidative stress index (OSI) was calculated by dividing TOS by TAC content and expressed in % [28].

FRAP content was determined spectrophotometrically by measuring the ferric reducing ability of samples and using 2,4,6-tripyridyl-s-triazine (TPTZ) [29]. The absorbance was measured at 593 nm, and FRAP was calculated from the calibration curve for FeSO_4_.

### 2.6. Proteins, Lipids, and DNA Oxidation Products

AGE content was determined spectrofluorimetrically by measuring characteristic fluorescence of AGE-derived compounds at 440/350 nm [30]. Immediately before the assay, the plasma samples were diluted 1 : 5 (*v* : *v*) in PBS (0.02 M, pH 7.0).

MDA concentration was assayed colorimetrically using the thiobarbituric acid reactive substances (TBARS) method with 1,3,3,3-tetraethoxypropane as a standard [31]. The absorbance was measured at 535 nm.

4-HNE and 8-OHdG concentrations were measured using commercial enzyme-linked immunosorbent assay (ELISA) according to the manufacturer's instructions (Cell Biolabs Inc., San Diego, CA, USA, and USCN Life Science). The absorbance was measured at 405 nm.

### 2.7. Statistical Analysis

The data were processed using Statistica 12.0 (StatSoft, Cracow, Poland) and GraphPad Prism 7 (GraphPad Software, La Jolla, USA). The statistical analysis was performed using unpaired Student's *t*-test and Pearson's correlation method (*p* set at 0.05). In the lack of normal distribution of the results, the nonparametric Mann–Whitney *U* test was used. The results were expressed as mean ± SD. The sample size was set based on a previously conducted pilot study (the power of the test was set at 0.9).

## 3. Results

### 3.1. General Characteristic of Rats

The energy intake in HFD rats was significantly higher (+27%) when compared to control animals. Therefore, despite the lowered food consumption (−26%), after 8 weeks of the experiment, the body weight as well as BMI were significantly increased (+21% and +27%, respectively) (Table 1). The increase of free fatty acids in plasma (+119%) of HFD-fed rats led to insulin resistance as fasting glucose and insulin levels as well as the HOMA-IR index were significantly higher (+70%, 12 times and 15 times, respectively) (Table 1). Increased amounts of ingested fats had no influence on the total protein concentration both in the cerebral cortex and hypothalamus.

### 3.2. Enzymatic and Nonenzymatic Antioxidants, Total Antioxidant/Oxidant Status, and Oxidative Damage Products in Plasma and Serum

Enzymatic antioxidant activity (GPx, CAT, and SOD-1) was significantly higher in the serum of HFD rats (+78%, +59%, and +46%); only GR activity remained unchanged when compared to the control (Table 2). Similarly, higher content of uric acid in the plasma of high fat diet-fed rats was observed (+44%). The total antioxidant/oxidant status and oxidative damage products (TAC, TOS, OSI, and FRAP) were also increased in animals which ingested high amounts of fats (+35%, +241%, +125%, and +66%, respectively). All of the estimated oxidative damage products (AGE, 4-HNE, MDA, and 8-OHdG) were significantly higher (+181%, +232%, +281%, and +30%, respectively) in the plasma of HFD animals (Table 2).

### 3.3. Prooxidant Brain Enzymes

**Figure 1:**
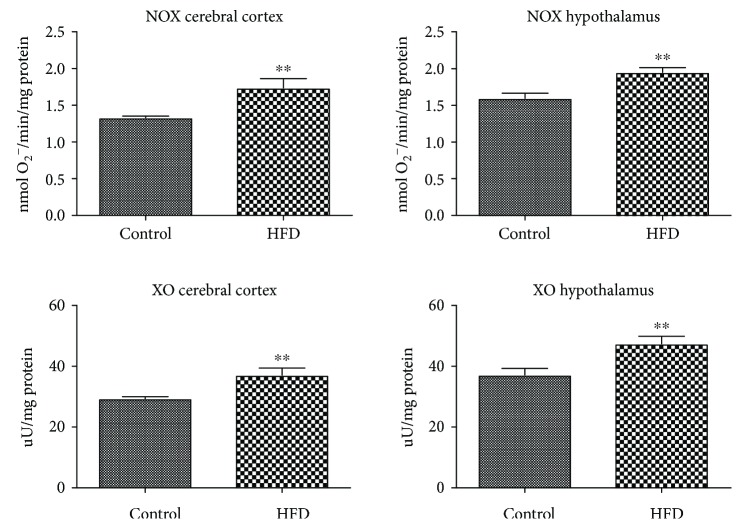
Prooxidant brain enzymes in the control and HFD-fed rats. NOX, NADPH oxidase; HFD, high fat diet; XO, xanthine oxidase. Differences statistically important at ^∗∗^*p* < 0.005.

The activity of NOX and XO was significantly increased in the animals fed HFD both in the cerebral cortex (+31% and +26%) and hypothalamus (+22% and +27%) (Figure 1).

### 3.4. Enzymatic and Nonenzymatic Brain Antioxidants

**Figure 2:**
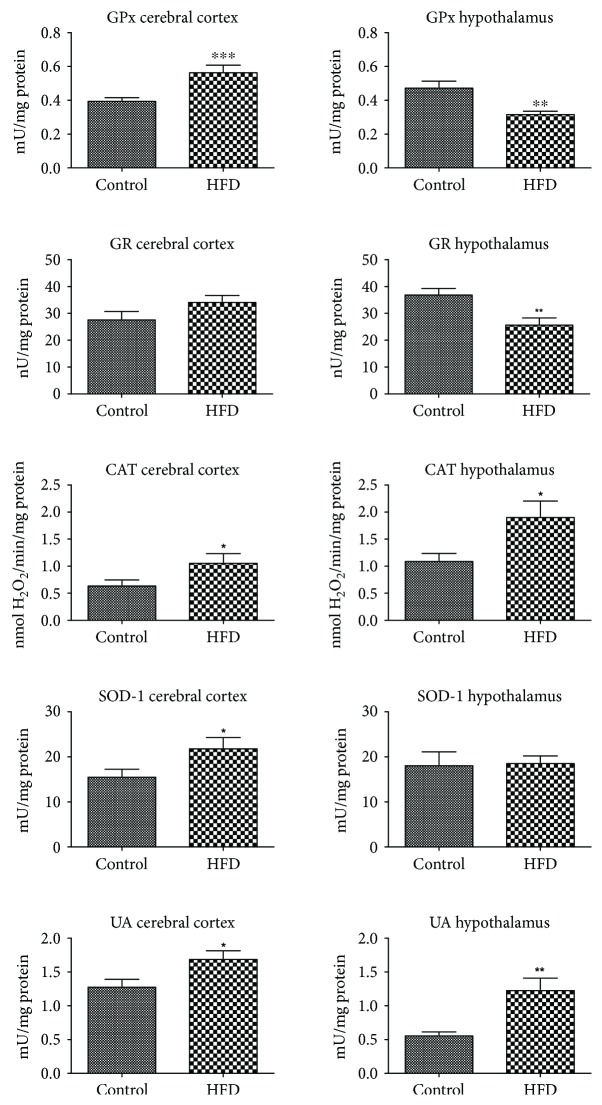
Enzymatic and nonenzymatic brain antioxidants in the control and HFD-fed rats. CAT, catalase; GPx, glutathione peroxidase; GR, glutathione reductase; HFD, high fat diet; SOD-1, Cu-Zn-superoxide dismutase-1; UA, uric acid. Differences statistically important at ^∗^*p* < 0.05, ^∗∗^*p* < 0.005, and ^∗∗∗^*p* < 0.0005.

The activity of enzymatic antioxidants (GPx, CAT, and SOD-1) in the cerebral cortex of the HFD-fed animals was markedly increased when compared to the control (+46%, +65%, and +40%, respectively) (Figure 2). On the other hand, in the hypothalamus of the HFD rats, only the activity of catalase was increased (+73%), whereas the activity of GPx and GR was significantly lower (−33% and −30% as compared to the control). Interestingly, uric acid content was increased in the cerebral cortex as well as in the hypothalamus (+32% and +117%) of the animals fed HFD (Figure 2).

### 3.5. Total Antioxidant/Oxidant Brain Status

**Figure 3:**
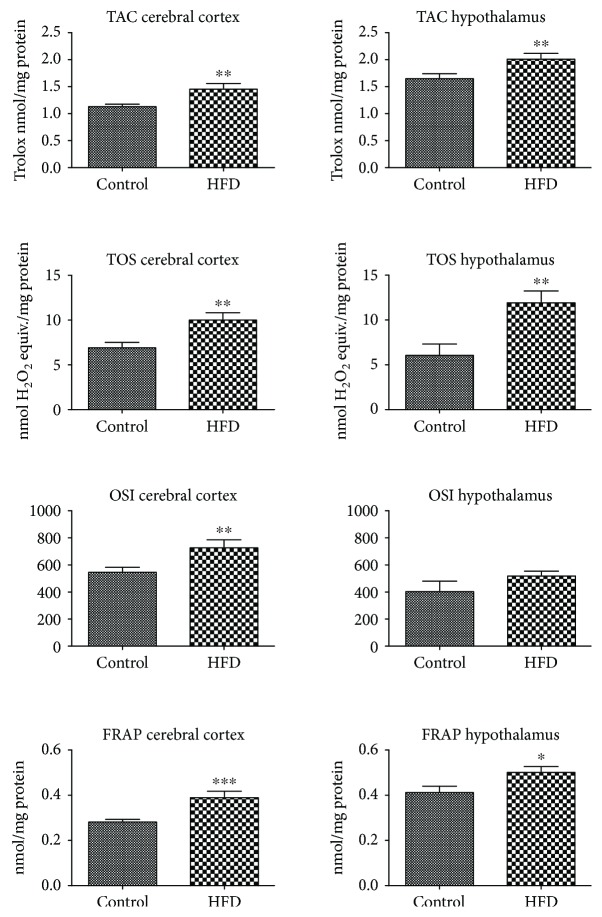
Total antioxidant/oxidant status in cerebral cortex and hypothalamus of the control and HFD-fed rats. FRAP, ferric reducing ability of sample; HFD, high fat diet; OSI, oxidative stress index; TAC, total antioxidant capacity; TOS, total oxidant status. Differences statistically important at ^∗^*p* < 0.05, ^∗∗^*p* < 0.005, and ^∗∗∗^*p* < 0.0005.

HFD leads to an increase in the antioxidant/oxidant status in both studied brain structures (except OSI in the hypothalamus). TAC, TOS, OSI, and FRAP were significantly higher in the cerebral cortex of HFD animals (+28%, +44%, +33%, and 34%, respectively). Similarly, in the hypothalamus, an increase in TAC, TOS, and FRAP was observed (+22%, 95%, and 21%) whereas OSI remained unchanged when compared to the control (Figure 3).

### 3.6. Oxidative Brain Damage Markers

**Figure 4:**
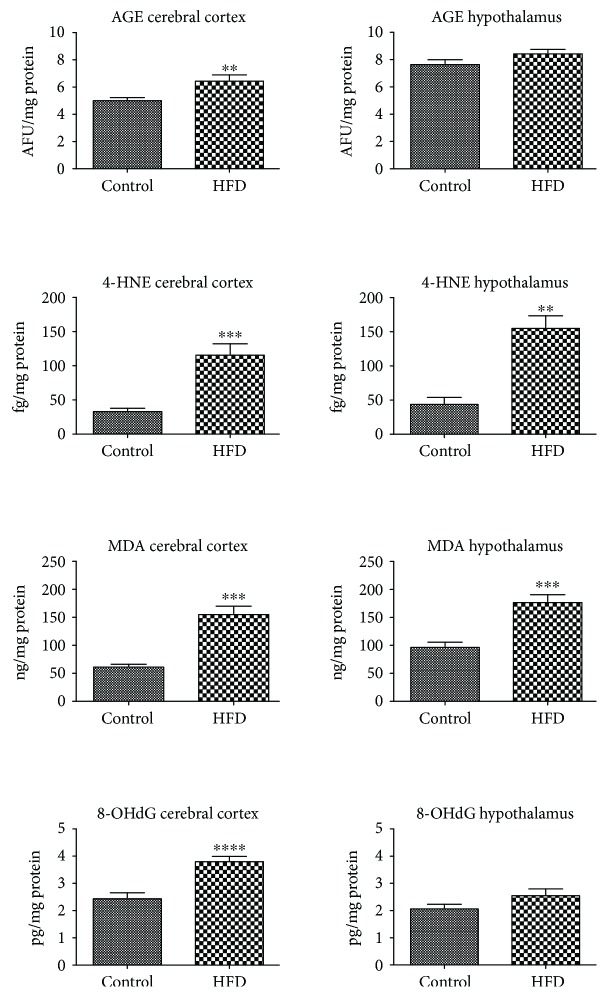
Oxidative damage to the cerebral cortex and hypothalamus of the control and HFD-fed rats. 4-HNE, 4-hydroxynonenal protein adducts; 8-OHdG, 8-hydroxy-2′-deoxyguanosine; AGE, advanced glycation end products; HFD, high fat diet; MDA, malondialdehyde. Differences statistically important at ^∗∗^*p* < 0.005, ^∗∗∗^*p* < 0.0005, and ^∗∗∗∗^*p* < 0.0001.

All of the oxidative damage markers (AGE, 4-HNE, MDA, and 8-OHdG) assayed in the cerebral cortex of HFD rats were significantly increased (+28%, +239%, +148%, and +55%) (Figure 4). In contrast, only 4-HNE protein adduct and MDA concentrations were markedly higher in the hypothalamus of high fat diet-fed animals (+248% and +82%).

### 3.7. Correlations

In the cerebral cortex of high fat-fed rats, we have shown a highly positive correlation between GPx activity and the TAC level (*r* = 0.86, *p* = 0.001), NOX and AGE (*r* = 0.9, *p* < 0.0001), and NOX and 4-HNE (*r* = 0.62, *p* = 0.01). In the HFD group, we have also demonstrated a positive correlation between the HOMA-IR index and AGE (*r* = 0.7, *p* = 0.05), as well as HOMA-IR and 4-HNE levels (*r* = 0.82, *p* = 0.001). There were no statistically significant correlations between the plasma/serum and brain OS biomarkers.

## 4. Discussion

This is the first study that compares both redox status, antioxidant defense, and oxidative damage between the hypothalamus and cerebral cortex of HFD-fed rats. We have demonstrated that chronic administration of HFD induces IR, which may be responsible for the redox imbalance, alternations in enzymatic and nonenzymatic brain antioxidants, and enhanced oxidative damage to the rats' cerebral cortex and hypothalamus. Moreover, we have observed a positive correlation between the brain oxidative damage and the HOMA insulin resistance index, which points at the involvement of systemic IR in the development of brain oxidative stress. Finally, although redox balance of both brain structures shifted towards the oxidative status, the intensity of oxidative damage was much greater in the rat's cerebral cortex.

Recently, a global epidemic of metabolic disorders caused by an improper diet and lack of physical activity is observed [32]. Excessive consumption of fat and cholesterol may lead to dyslipidemia, obesity, hyperglycemia, hyperinsulinemia, and whole-body IR [5]. Therefore, it is not surprising that the applied model of chronic (8 weeks) HFD resulted in higher plasma free fatty acids, elevated fasting glucose and insulin, and reduced insulin sensitivity indicated by the higher HOMA-IR index. Based on the available literature and diagnostic criteria of IR [32, 33], we have confirmed the occurrence of IR in all the HFD-fed animals. In this group, we have also observed a significant increase in body weight and body mass index (BMI), as well as general (plasma/serum) oxidative stress, which is consistent with the previous reports on the animal models of IR [2, 32].

It is beyond question that the main free radical source in the brain is the increased oxidation of glucose and free fatty acids in mitochondria [32, 34]. Of all the by-products generated during mitochondrial processes, superoxide anions (O_2_^−•^) are formed in the largest quantities [34]. Up to 90% of ROS is produced during oxidative phosphorylation, while enhanced oxidation of energy substrates can further increase the formation of free radicals in the cell [2, 32]. Indeed, when caloric intake is enhanced, the number of electrons supplied to the respiratory chain is increased and generates more O_2_^−•^. The production of O_2_^−•^ is also catalyzed by the enzymes from the oxidoreductase class including NADPH oxidase (NOX) and xanthine oxidase (XO) [35]. The results of the presented study indicate a higher activity of ROS-generating enzymes (NOX and XO) in the hypothalamus and cerebral cortex of HFD-fed animals. Referring to the previous research, exposure to HFD stimulates inflammatory signaling in macrophages and adipocytes, which results in the NOX activation and increased production of proinflammatory cytokines (e.g., IL-1*β*, IL-6, and TNF*α*) [36]. It is well known that NOX and XO can dramatically increase a rapid consumption of oxygen during an oxidative respiratory burst in mitochondria [34]. They can also stimulate adipocytes to release MCP-1 (monocyte chemoattractant protein-1), which promotes the transformation of monocytes to macrophages and intensifies inflammation [36]. Bearing in mind that the increased expression of cytokines (e.g., IL-1*β*, IL-6, and TNF*α*), chemokines, and proinflammatory enzymes (e.g., COX-2 and iNOS) have been reported in the IR brain [37, 38], the enhanced activity of NOX and XO may not only be an important source of ROS but can also induce an inflammatory response in the hypothalamus and cerebral cortex of HFD-fed rats.

To prevent the harmful effects of ROS, the brain has developed specialized antioxidative systems including enzymatic (SOD, CAT, and GPx) and nonenzymatic (UA and reduced glutathione) brain antioxidants. However, the total antioxidant effect is not a simple sum of individual antioxidants separately. The ability to scavenge free radicals and/or reduce oxidative damage in the cell depends on the mutual interactions of all brain antioxidants [35]. It is well known that a very important parameter to assess the redox balance in biological systems is the total antioxidant capacity (TAC) and the total oxidant status (TOS) [26]. TAC determines the overall ROS scavenging ability, whereas TOS can be defined as the total level of oxidants (ROS) in the sample [26, 27, 35]. Therefore, despite the increase in some antioxidative mechanisms (↑CAT and ↑UA) and the decrease of other antioxidant enzymes (↓GPx and ↓GR), the ROS removal efficiency of the hypothalamus is more severe in HFD-fed rats (↑TAC and ↑FRAP) in comparison to the control. In our study, we have also showed enhanced antioxidant efficiency in the cerebral cortex of HFD-fed rats (↑GPx, ↑CAT, ↑SOD-1, ↑UA, ↑TAC, and ↑FRAP). Therefore, the elevated activity/level of antioxidants indicates an adaptive brain response to the increased production of ROS under IR pathology. It is undeniable that the strengthening of the antioxidant's barrier is the most important mechanism for limiting the formation of ROS and regulating their activity. However, do the antioxidant systems prevail over the free radical reactions? For the quantitative assessment of redox homeostasis disorders, we have used the oxidative stress index (OSI; TOS to TAC ratio), which is referred to as the “gold indicator of oxidative stress” [28]. We have demonstrated that in the HFD-fed rats, the level of oxidants (TOS) outweighs the antioxidant defense mechanisms (TAC), which leads to a shift in the redox balance in favor of the oxidation reactions. Significantly higher OSI in the cerebral cortex of HFD-fed rats suggests a greater extent of oxidative processes in comparison to the hypothalamus. Additionally, the disturbances in oxidant/antioxidant status have also been reported in the blood of HFD-fed animals (↑TAC, ↑TOS, ↑OSI, and ↑FRAP).

Redox abnormalities observed in the study may have consequences in the cellular manifestations of diet-induced IR. Despite the enhanced activity of the brain and blood antioxidative systems, we have shown an increase in the level of oxidized biomolecules (↑AGE, ↑4-HNE, ↑MDA, and ↑8-OHdG) both in the brain and plasma of HFD-fed animals. It should be noted that the brain is a significant target for oxidative stress due to its increased oxygen consumption, limited detoxification mechanisms, and high content of prooxidative metal ions (mainly iron and copper) [39, 40]. Under these conditions, ROS may alter the cellular components resulting in the enhanced peroxidation of the membrane lipids. Indeed, we have demonstrated significantly higher levels of lipid oxidation products (↑4-HNE and ↑MDA) in both the hypothalamus and cerebral cortex of HFD-fed rats. It is well known that these compounds, mainly 4-hydroxynonenal (4-HNE), can alter the fluidity and integrity of cells and can also react with proteins and nucleic acids, resulting in further oxidative damage [14]. 4-HNE is also implicated with ATP depletion, impaired glucose transport, and oxidative damage to the active centres of enzymes in the hippocampal and cortical neurons [41, 42]. In our study, in addition to the increased concentration of 4-HNE in both brain structures of HFD-fed rats, we also observed an increase in MDA levels. Malondialdehyde shows mutagenic and carcinogenic effects that can affect the brain cell's proliferation and lead to the apoptotic cell death [34, 40]. In addition, it has been shown that MDA and 4-HNE may increase permeability of the blood-brain barrier (BBB) [40, 43], which may be one of the causes of cerebral neurodegeneration. Deleterious consequences of lipid peroxidation on the brain may also be the induction of proinflammatory enzymes in macrophages [40] and thus stimulation of inflammation. In our study, this may be confirmed by the positive correlation between the 4-HNE level and the NOX activity in the cerebral cortex of HFD-fed rats.

The effect of free radical interactions is not only damage to the lipids but also oxidation of proteins and nucleic acids (DNA or RNA). Oxidative DNA damage is particularly dangerous for the brain because it can cause genetic instability and lead to neuronal cell death via apoptosis or necrosis pathways [7]. In our study, we showed significantly higher concentrations of DNA injury marker 8-OHdG in the cerebral cortex and plasma of HFD-fed rats vs. the control group. Both nuclear and mitochondrial DNA can undergo oxidative damage, which can result in a decrease in the ATP concentration (required for the sodium-potassium ATPase) and other transport proteins in the cell [42]. In our study we have also observed the elevated content of AGE (products of glycation and oxidation by reducing sugar) in the cerebral cortex of rats within the study group. It is believed that the increase in nonenzymatic glycation of proteins and lipids is the main cause of neurodegeneration and cerebral changes caused by aging [44]. Higher AGE levels have also been observed in many metabolic diseases, particularly in the hyperglycemic conditions [16, 45]. Binding of AGE to a specific receptor (RAGE) activates many transcription factors and inflammatory signaling pathways including NFΚB, MAP-kinase, NJK, and p21RAS [16, 46]. The presence of RAGE receptors has been demonstrated on the surface of the endothelial cells, cardiomyocytes, dendritic cells, monocytes/macrophages and also the CNS neurons [16]. What is important, AGE may also increase the production of ROS by inducing the activity of prooxidant enzymes, especially NADPH oxidase and XO [14]. Additionally, oxidized proteins tend to form aggregates resistant to degradation, which favors the accumulation of altered proteins in cells and intensifies inflammation [32]. This seems to explain the positive correlation between the AGE content and NOX activity in the cerebral cortex of HFD-fed rats. Moreover, it suggests that glycooxidative damage to cells is directly responsible for the progression of chronic complications of IR and T2DM. It has been shown that serum AGE concentration correlates with the IR level assessed by the HOMA-IR index [44, 45]. In our study, we have reported a highly positive correlation between the HOMA-IR index and the brain oxidative damage to the proteins (AGE) and lipids (4-HNE). This suggests that central IR may affect the development of cerebral complications of the disease. However, we did not observe any relationship between the blood and brain oxidative stress biomarkers, which indicates the different nature of oxidative damage at the central and local level. This may also indicate that oxidative modification products are formed directly in the brain and do not pass from the circulation through the BBB. Similarly, we did not notice any correlation between the antioxidant defense and redox homeostasis indicators. Thus, the processes taking place in the brain occur independently from the general (plasma/serum) oxidative stress.

An important conclusion from our study is the fact that the cerebral cortex is more strongly affected by oxidative stress than the hypothalamus. Increased concentrations of protein (↑AGE) and DNA (↑8-OHdG) oxidative modifications have been observed only in the cerebral cortex of HFD-fed animals. Although our experiment does not explain the differences between the different brain sensitivity to cellular oxidative stress, it can be assumed that the greater vulnerability to oxidation of the cerebral cortex may be caused by its greater ability to accumulate prooxidative metal ions [47]. Under these conditions, significant amounts of the hydroxyl radicals can be formed. It has been shown that even a slight increase in the transition metals content (e.g., Fe^2+^, Cu^2+^, Co^2+^, V^2+^, and Cr^2+^) may significantly enhance OH^•^ production in the brain [39, 40]. It is well known that OH^•^ is able to react with all adjacent biomolecules. However, membrane lipids are the most exposed to OH^•^ and other ROS [34]. Cellular lipids are also a rich source of polyunsaturated fatty acids (PUFAs) that are particularly susceptible to oxidation via oxidative stress [39, 48]. Therefore, this is not surprising that the hypothalamic oxidative damage has only been reported to the lipid molecules.

In our study, the elevated rate of oxidative processes in the IR cerebral cortex is also demonstrated by an increase in OSI levels, suggesting higher production of ROS in comparison to the hypothalamus. The reason for the observed differences may also be the changes in brain bioenergetics and mitochondrial functioning. Indeed, it has been shown that the cerebral cortex and hypothalamus are characterized by different energy metabolisms which additionally changes with age [49]; however, the presented issue requires further research and observations.

When analyzing the results of our work, attention should also be paid to its limitation. Firstly, we evaluated only selected, though the most commonly used, biomarkers of redox homeostasis and oxidative stress. Secondly, the observed changes in the oxidant-antioxidant balance may be the result of not only IR but also other disturbances induced by HFD (e.g., obesity, hyperinsulinemia, hyperglycemia, or metabolic syndrome). In addition, we must not ignore the fact that oxidative stress may be the cause of diet-induced IR. We believe that only kinetic studies can explain this issue. However, it should be borne in mind that this is the first study in which the hypothalamic and cortical oxidative stresses were compared in HFD-fed animals. We also characterized the redox balance at the systemic and local levels, which is an undeniable advantage of our work.

## Figures and Tables

**Table 1:** Effect of 8-week HFD on body weight, BMI, glucose, insulin, HOMA-IR, food and energy intake, and total protein concentration.

	C (*n* = 8)	HFD (*n* = 8)
Final body weight (g)	312.2 ± 15.2	378.1 ± 17.6^∗^
BMI (g/cm^2^)	0.52 ± 0.1	0.66 ± 0.3^∗^
Fasting glucose (mg/dL)	99.8 ± 4.5	169.6 ± 15.1^∗^
Fasting insulin (*μ*U/mL)	4.7 ± 0.3	54.6 ± 4.1^∗^
HOMA-IR index	1.4 ± 1.2	21.3 ± 1.6^∗^
Plasma FFA (*μ*mol/L)	79.5 ± 10.7	174.3 ± 10.2^∗^
Food consumption (g/day)	23.2 ± 0.8	17.1 ± 0.6^∗^
Energy intake (kJ/day)	266.2 ± 3.2	338.9 ± 5.1^∗^
Cerebral cortex total protein content (*μ*g/mL)	2515.0 ± 94.9	2159.0 ± 44.2
Hypothalamus total protein content (*μ*g/mL)	1892.0 ± 121.7	1616.0 ± 66.4

FFA, free fatty acids; HOMA-IR, homeostatic model assessment of insulin resistance; HFD, high fat diet. Differences statistically important at ^∗^*p* < 0.05.

**Table 2:** Effect of 8-week HFD on enzymatic and nonenzymatic antioxidants, total antioxidant/oxidant status, and oxidative damage products in the rat's plasma and serum.

	C (*n* = 8)	HFD (*n* = 8)
GPx (mU/mg protein)	0.9 ± 0.09	1.6 ± 0.1^∗^
GR (nU/mg protein)	55.3 ± 0.7	56.55 ± 0.2
CAT (nmol H_2_O_2_/min/mg protein)	3.9 ± 0.2	6.2 ± 0.2^∗^
SOD-1 (mU/mg protein)	29.4 ± 1.3	42.9 ± 1.6^∗^
UA (*μ*g/mg protein)	3.4 ± 0.3	4.90 ± 0.3^∗^
TAC (Trolox nmol/mg protein)	9.6 ± 0.2	13.0 ± 0.9^∗^
TOS (nmol H_2_O_2_ equiv./mg protein)	36.9 ± 1.9	126.0 ± 5.0^∗^
OSI	433.5 ± 32.7	976.0 ± 38.2^∗^
FRAP (nmol/mg protein)	1.15 ± 0.3	1.91 ± 0.2^∗^
AGE (AFU/mg protein)	29.7 ± 0.6	83.6 ± 0.2^∗^
4-HNE (fg/mg protein)	51.5 ± 5.5	170.8 ± 10.6^∗^
MDA (ng/mg protein)	70.2 ± 8.2	267.4 ± 15.5^∗^
8-OHdG (pg/mg protein)	6.6 ± 0.3	8.6 ± 0.3^∗^

4-HNE, 4-hydroxynonenal protein adducts; 8-OHdG, 8-hydroxy-2′-deoxyguanosine; AGE, advanced glycation end products; CAT, catalase; FRAP, ferric reducing ability of sample; GPx, glutathione peroxidase; GR, glutathione reductase; HFD, high fat diet; MDA, malondialdehyde; OSI, oxidative stress index; SOD-1, Cu-Zn-superoxide dismutase-1; TAC, total antioxidant capacity; TOS, total oxidant status; UA, uric acid. Enzymatic antioxidants (GPx, GR, CAT, and SOD-1) were determined in serum whereas other markers were assayed in plasma. Differences statistically important at ^∗^*p* < 0.05.

## Data Availability

All of the data used to support the findings of this study are included within the article.
